# Genomic Surveillance of SARS-CoV-2 Variants That Emerged in South and Southeast Asia during Early 2022

**DOI:** 10.3390/v15061355

**Published:** 2023-06-12

**Authors:** Qiong Yu, Xi Tong, Li Zuo, Xinyu Tao, Zhonghui Xu, Xiaocui Li, Haizhou Liu, Wuxiang Guan, Di Liu, Haibin Liu, Fang Huang, Lijia Jia

**Affiliations:** 1 Key Laboratory of Special Pathogens and Biosafety, Center for Biosafety Mega-Science, Wuhan Institute of Virology, Chinese Academy of Sciences, Wuhan 430071, China; yuqiong169@163.com (Q.Y.); tongxi21@mails.ucas.ac.cn (X.T.); 18942561396@163.com (L.Z.); taoxinyu221@mails.ucas.ac.cn (X.T.); liuhz@wh.iov.cn (H.L.); guanwx@wh.iov.cn (W.G.); liud@wh.iov.cn (D.L.); hbliu@wh.iov.cn (H.L.); 2 University of Chinese Academy of Sciences, Beijing 100045, China; 3 Livzon Bio Inc., Zhuhai 519045, China; xuzhonghui@livzon.cn (Z.X.); lixiaocui@livzon.cn (X.L.); 4 Hubei Jiangxia Laboratory, Wuhan 430200, China

**Keywords:** SARS-CoV-2, variant, genomic epidemiology, evolutionary dynamics

## Abstract

The continuously emerging new variants of severe acute respiratory syndrome coronavirus 2 (SARS-CoV-2) have made the global coronavirus disease 2019 (COVID-19) pandemic unpredictable. Since the beginning of the pandemic, densely populated South and Southeast Asia have suffered great losses due to multiple COVID-19 surges because of vaccine and other medical resource shortages. Therefore, it is crucial to closely monitor the SARS-CoV-2 epidemic and to understand the evolutionary and transmission characteristics of SARS-CoV-2 in these regions. Here, we document the evolution of epidemic strains in the Philippines, Pakistan, and Malaysia from late 2021 to early 2022. Our results confirmed the circulation of at least five SARS-CoV-2 genotypes in these countries in January 2022, when Omicron BA.2, with a detection rate of 69.11%, replaced Delta B.1.617 as the dominant strain. Single-nucleotide polymorphism analysis indicated the distinct evolutionary directions of the Omicron and Delta isolates, with *S*, *Nsp1*, and *Nsp6* genes potentially playing a significant role in the host adaptation of the Omicron strain. These findings are able to provide insights for predicting the evolutionary direction of SARS-CoV-2 in terms of variant competition, developing multi-part vaccines, and to support the evaluation and adjustment of current surveillance, prevention, and control strategies in South and Southeast Asia.

## 1. Introduction

Since the first coronavirus disease (COVID-19) outbreak began in December 2019, more than 760 million confirmed cases and nearly 7 million deaths have been reported worldwide [1], making COVID-19 one of the deadliest epidemics in human history. In response to the outbreak, many countries have taken restrictive measures, such as wearing face masks and conducting contact tracing, over the past two years, and vaccines have been developed at an unprecedented rate [2,3,4]. However, the continuous emergence of severe acute respiratory syndrome coronavirus 2 (SARS-CoV-2) variants has increased the risk of transmission, reduced the effectiveness of neutralizing antibodies and vaccination, and caused outbreaks to reoccur in many parts of the world. More than 1000 SARS-CoV-2 variants have been identified, among which Alpha, Beta, Gamma, Delta, and Omicron have been deemed to be variants of concern that circulate globally by the World Health Organization [5,6]. To make matters worse, SARS-CoV-2 continues to rapidly evolve, creating uncertainty about the new wave of outbreaks that the world is experiencing.

As the world’s most populous regions, South and Southeast Asia have witnessed remarkable economic growth and huge market potential in recent years. However, countries in these regions have suffered a lot due to COVID-19 because of vaccine and other medical resource shortages [7]. As of September 2021, 11,324,390 confirmed cases had been reported in Southeast Asia, including 249,529 deaths, with a fatality rate of 3.3%, which is significantly higher than the global average [8]. Three major prevalent strains were responsible for this result: Alpha, Beta and Delta (respectively corresponding to Pango lineages B.1.1.7, B.1.351, and B.1.617.2) [9], among which the Delta variant may be more than twice as transmissible as the original strain is [10]. A new, more infectious SARS-CoV-2 variant, Omicron (B.1.1.529), was discovered in South Africa on 24 November 2021 and was classified as the newest variant of concern, which later proved to have a 2.7–3.7 times higher infection rate than the Delta strain has [11,12]. In the context of a new wave of variants, it is essential to closely monitor the SARS-CoV-2 epidemic in South and Southeast Asia to allow timely interventions to reduce the risks of mass morbidity and mortality.

In this study, we tracked the prevalence of SARS-CoV-2 strains in the Philippines, Pakistan, and Malaysia from late 2021 to early 2022 based on a clinical trial conducted by LivzonBio, Inc. It was found that, such as in other regions of the world at that time, the predominant epidemic strain detected in these three countries was characterized by a transition from Delta to Omicron over time, with the proportion of Omicron infections increasing from 40% to 86.8% in only one month (from December 2021 to January 2022). We also report genome-wide variation hotspots in Omicron isolates. We expect our study will provide ideas for predicting the evolutionary direction of SARS-CoV-2 in terms of variant competition and for developing multi-part vaccines and provide data to support the assessment and adjustment of current surveillance, prevention, and control strategies in South and Southeast Asia.

## 2. Materials and Methods

### 2.1. Clinical Sample Collection

The clinical samples (*n* = 241) used in this study were obtained from a phase III clinical trial of a recombinant SARS-CoV-2 fusion protein vaccine (V-01) that was globally produced by LivzonBio (Zhuhai, China). Throat swabs were collected from patients that were identified as being SARS-CoV-2 positive via real time quantitative reverse transcription PCR (qRT-PCR) in Malaysia, Pakistan, and the Philippines from October 2021 to January 2022. All throat swab samples from COVID-19 patients were subjected to whole-genome sequencing. Based on the progress of the clinical trials, we obtained 92 genome sequences, with a coverage rate of over 70%, which were used for downstream analysis. Patients from the Philippines were unvaccinated prior to enrollment, whereas those from Pakistan and Malaysia received two COVID-19 vaccine doses.

### 2.2. RNA Extraction and qRT-PCR Detection

Total RNA was extracted using the NA96 automated nucleic acid extraction system and a nucleic acid extraction kit (Zhuhai Livzon Diagnostics Inc., Zhuhai, China). Briefly, nucleic acids were isolated from the specimens under the treatment with extraction reagent A (containing strong protein denaturants) and proteinase K. Then, the mixtures were incubated with magnetic beads for RNA purification. Bound RNA was eluted and subjected to the following qRT-PCR detection. qRT-PCR target ORF1ab gene and the N gene of SARS-CoV-2 were used to detect and quantify the viral RNA obtained from clinical samples using the Novel Coronavirus (2019-nCoV) Nucleic Acid Diagnostic Kit (Real-time PCR Fluorescence Probing) according to the manufacturer’s instructions (Zhuhai Livzon Diagnostics Inc., Zhuhai, China). The viral load was quantified using the cycle threshold (Ct) values.

### 2.3. Library Preparation and Next-Generation Sequencing

After DNA removal via DNase I digestion, the concentration of total RNA was measured using the Qubit RNA HS Analysis Kit (Thermo Fisher Scientific, Waltham, MA, USA). Total RNA was reverse transcribed to generate the first-strand cDNA with the ATOPlex RNA Multiplex PCR Amplification Kit V3.0 (MGI, Shenzhen, China). DNA libraries were prepared using the MGIEasy Fast PCR-FREE Enzyme Library Preparation Kit (MGI, Shenzhen, China) according to the manufacturer’s instructions. DNA libraries were quantified using the Qubit^®^ dsDNA HS Assay Kit (Thermo Fisher Scientific, Waltham, MA, USA) (the concentration was ≥0.8 ng/μL). DNA libraries were further prepared for DNA nanoball (DNB) sequencing using the DNBSEQ One Step DNB Preparation Kit (MGI, Shenzhen, China) according to the manufacturer’s instructions. DNB libraries were sequenced on an ultra-high-throughput MGISEQ-2000RS platform (MGI, Shenzhen, China) using a single-end 100 nt sequencing strategy, generating an average of 1 Gb of sequencing data for each sample.

### 2.4. Genome Assembly and Phylogenetic Analysis

After next-generation sequencing, raw data were processed, and the SARS-CoV-2 genomes were assembled using the metarget COVID software (v.3.1; MGI). Based on the globally subsampled SARS-CoV-2 dataset created by Nextstrain (https://nextstrain.org/ncov/gisaid/global/all-time (accessed on 13 January 2023)) at the start of the pandemic, we selected 1440 reference sequences with clear sample information from GISAID (https://www.epicov.org/ (accessed on 13 January 2023)) and combined them with the 92 sequences obtained in this study to construct a phylogenetic tree. We used MAFFT v.7.475 [13] to perform multiple sequence alignments on all sequences, IQ-TREE v.2.0.3 [14,15] to construct a maximum-likelihood phylogenetic tree, and ggtree v.3.6.2 [16] for annotation.

UShER’s pre-processed mutation-annotated tree object for public SARS-CoV-2 sequences obtained by 31 January 2022 was used as a reference dataset (https://hgdownload.soe.ucsc.edu/goldenPath/wuhCor1/UShER_SARS-CoV-2/2022/01/31/ (accessed on 24 February 2023) to infer the relations of isolates to reference strains. VCF files of the 92 isolate sequences were uploaded to UshER (v.0.6.2), and the isolates were placed on the appropriate clade of the phylogenic tree [17]. Information about the strain closest to each isolate was determined using the subtree size parameter.

### 2.5. Single-Nucleotide Polymorphism (SNP) Analysis

An in-house Perl script (https://github.com/zer0liu/bioutils/tree/master/snp) was used to identify SNPs. The earliest strain, Wuhan-Hu-1 (GenBank accession No.: NC_045512.2), was used as a reference genome. SNP type, distribution, and density were determined using the ggplot2 package v.3.4.1 in R v.4.2.2 [18]. Fisher’s exact test was used to assess whether SNP distributions were significantly different among the coding regions (CDSs).

## 3. Results

### 3.1. Cohort of COVID-19 Patients in South and Southeast Asia Sampled during Winter from Late 2021 to Early 2022

**Figure 1:**
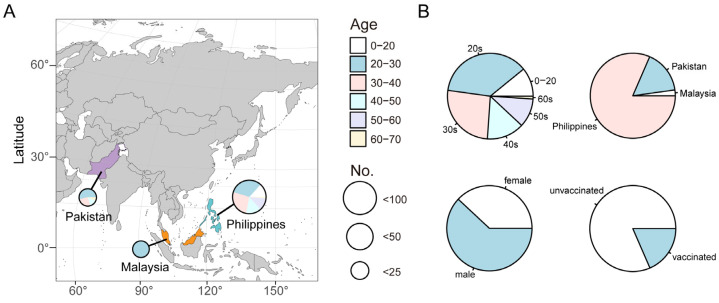
Geographical distribution and classification characteristics of COVID-19 patients. (**A**) Clinical sampling map. The size of each pie chart represents the sample size and the color represents the proportion of patients in different age groups. (**B**) Pie charts showing the distribution of patients by age, country, gender, and status of vaccination against COVID-19.

From October 2021 to January 2022, whole-genome sequencing was performed on V01-III clinical COVID-19-positive pharyngeal swabs taken from patients and received from LivzonBio, Inc. Based on the progress of clinical trial, we obtained 92 genome sequences, with a coverage of over 70%, which ensures quality control for downstream analysis (Supplementary Table S1). As shown in Figure 1A, the COVID-19 patient samples analyzed in this study were from three countries: the Philippines, Pakistan, and Malaysia. All participants were adults of Asian descent that were above 18 years of age. The COVID-19 patients’ characteristics and sampling distributions are shown in Figure 1B. The median age at inclusion visit was 31 years (18–64 years), with 10.87% of participants being in the age group 18–20, 36.96% being in the age group 20–30, 26.09% being in the age group 30–40 years, 14.13% being in the age group 40–50 years, 10.88% being in the age group 50–60 years, and 1.09% being in the age group 60–70 years. Most participants were 20–40 years (63.04%), implying that this study focused on young people. Of the total pharyngeal swab results, 2.17% were from Malaysia, 16.30% were from Pakistan, and 81.52% were from the Philippines. Among all the subjects, 38.04% were female, and 61.96% were male. Notably, 18.48% of the participants had received two doses of COVID-19 vaccines prior to enrollment, and they were from Pakistan and Malaysia. The remaining 81.52% of participants were from the Philippines and had not received any vaccinations before enrollment. These basic data provided important references for the subsequent data analysis.

We determined the viral load in the 92 clinical samples and found that the Ct values of patients who received two vaccine doses were higher than those of the unvaccinated patients (Figure S1), suggesting that vaccination against COVID-19 reduces the viral load in positive patients to some extent. No significant differences in Ct values were found among the vaccinated patients stratified by age or gender.

### 3.2. Genotyping and Phylogenetic Analyses of SARS-CoV-2

**Figure 2:**
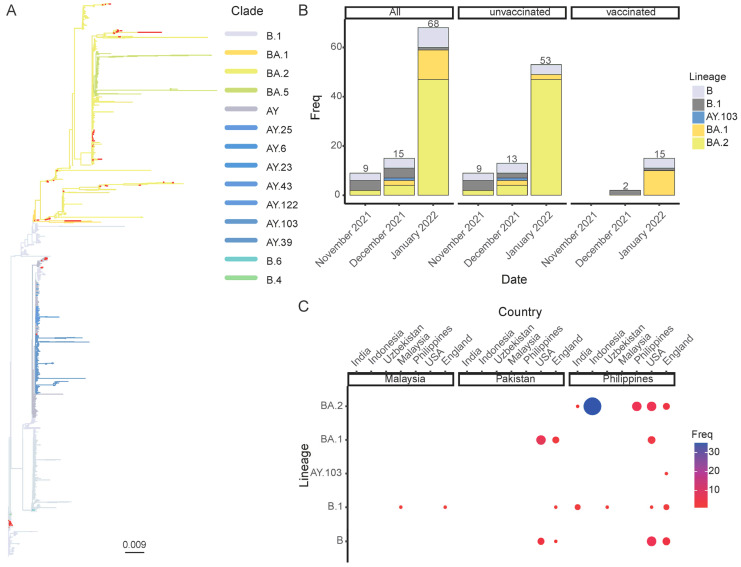
Phylogenetic analysis of SARS-CoV-2. (**A**) Maximum-likelihood phylogenetic tree of SARS-CoV-2 based on whole genomes. Using the NC_045512.2 reference sequence as the root, the tree was constructed using a dataset of 1440 SARS-CoV-2 genomes from GISAID as the reference. The color of the branches represented different genotypes, with the 92 isolates from this study shown in red. (**B**) Month-by-month prevalence characteristics of SARS-CoV-2 from November 2021 to January 2022. (**C**) Closest neighbors of the isolates from this study.

To further investigate the genotypes and evolutionary characteristics of the SARS-CoV-2 isolates, we integrated isolates from GISAID prior to 26 November 2022 as a reference dataset with isolates obtained in this study to construct a phylogenetic tree based on the whole viral genomes. Overall, the phylogenetic tree exhibited an unbalanced ladder structure, indicating that the evolution of SARS-CoV-2 was subjected to continuously strong immune selection pressure (Figure 2A). The genotyping results revealed that out of the 92 samples, 67 cases were identified as Omicron (15 cases of BA.1 and 52 cases of BA.2) and 10 cases were identified as Delta (9 cases of B.1 and 1 case of AY.103) (Figure 2A). The remaining 15 cases were classified as B.

We noticed that from November 2021 to January 2022, the detection rate of the Delta variant gradually decreased over time, whereas that of Omicron rapidly increased, reaching 86.76% in January 2022, of which BA.1 accounted for 17.65%, BA.2 accounted for 69.11%, and Delta accounted for only 1.47% at this time (Figure 2B). These findings suggested that during this period, the predominant epidemic variant in these regions transitioned from Delta to Omicron, which is consistent with the global evolution of SARS-CoV-2. Furthermore, we found that there were more cases of BA.1 infections than of those of BA.2 infections in the vaccinated group (Figure 2B). However, more data are needed to determine whether this suggests that the BA.1 strain has higher vaccine-escape capability.

To explore the cross-regional transmission of existing epidemic strains in Malaysia, the Philippines, and Pakistan, we utilized Usher to identify the nearest neighbors of the study isolates. The results showed that the BA.1 isolates from Malaysia were closely related to a strain circulating in England, and the BA.1 isolates from Pakistan and the Philippines were closely related to a strain found in the United States of America (USA). Moreover, the BA.2 strain in the Philippines presented the most similarity with a strain isolated from Indonesia (Figure 2C). Delta and Omicron BA.1 strains in all three countries were closely related to the strains circulating in England and USA, whereas Omicron BA.2 strains from the Philippines clustered with several regional isolates, indicating multiple sources of transmission.

### 3.3. Genomic Diversity of SARS-CoV-2 Isolates

**Figure 3:**
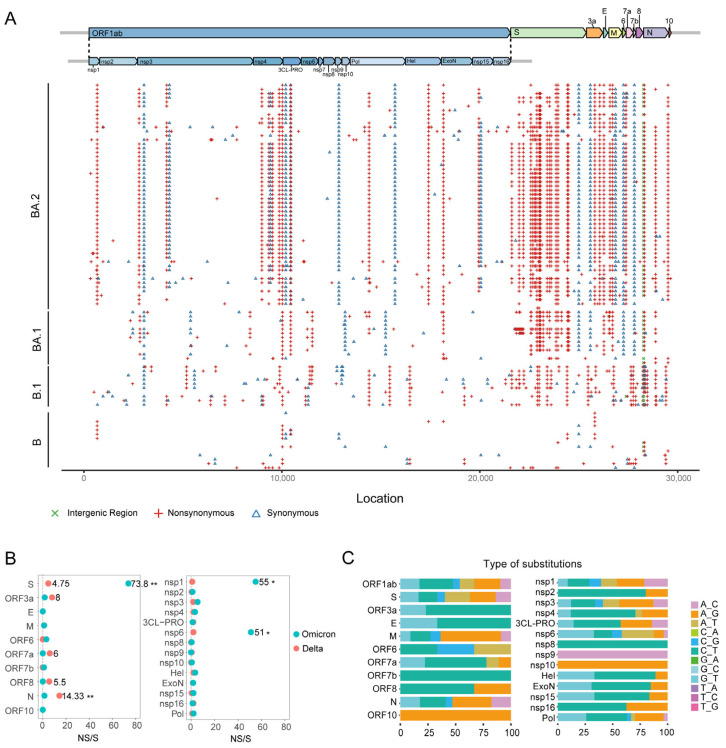
Mutation characteristics of the SARS-CoV-2 genome. (**A**) SNP distributions in the SARS-CoV-2 genomes. NS substitutions are indicated in red, S substitutions are indicated in blue, and SNPs are indicated in intergenic regions in green. (**B**) NS/S ratio of each ORF in the Delta and Omicron strains. (**C**) Statistics of SNP types. Fisher’s exact test was used to assess the significance of differences between ORFs. * *p* < 0.05; ** *p* < 0.01.

By taking NC_045512.2 as the reference genome, altogether, we identified 1035 SNPs in the isolates in this study, 943 of which were located in CDS regions, and the other 92 were in intergenic and untranslated regions. Among the SNPs in CDS regions, 819 were non-synonymous (NS) substitutions, and only 132 were synonymous (S) substitutions (Figure 3A), indicating that the SARS-CoV-2 genome was under positive selection pressure, likely due to the adaptation to the host environment and immune responses.

Next, we filtered out SNPs with an allelic mutation frequency of <75% to reduce the impact of atypical mutations in a few isolates and retained only high-frequency mutations. The high-frequency substitution sites in the Omicron strain were mainly concentrated in the *S* gene (A22578G, G22882T, A22992G, A23013C, A23040G, A23055G, A23063T, C23075T, C23525T, A23854C, G23948T, A24424T, and A24469T), whereas the high-frequency substitution sites in the Delta strain were more evenly distributed among the *ORF1ab*, *S*, *ORF3ab*, *M*, *ORF7a*, and *N* genes (C14408T, A15451G, C16466T, G22917T, A23403G, A24410G, C25469T, C26767T, C27638T, C27752T, A28461G, A28881C, and G29402T) (Figure 3A, Table 1). After normalizing the SNP distributions according to open reading frame (ORF) length, we found that most genes exhibited a higher NS substitution rate than the level of the S substitution rate, indicating that SARS-CoV-2 rapidly mutated to adapt to the host. It is worth noting that in the Delta strain, the NS/S ratio of *N* was significantly higher than that of other ORFs (Fisher’s exact test, *p* < 0.01), whereas the NS/S ratio of *Nsp2* was lower than that of other ORFs (Fisher’s exact test, *p* < 0.01). In contrast, in the Omicron strain, the NS/S ratios of *S*, *Nsp1*, and *Nsp6* were significantly higher than those of other ORFs (Fisher’s exact test, *p* < 0.05), whereas the N/S ratios of *M*, *N*, *ExoN*, and *Pol* were significantly lower than those of other ORFs (Fisher’s exact test, *p* < 0.01) (Figure 3B). These findings provide insights into the evolutionary dynamics of SARS-CoV-2 and suggest that the Delta and Omicron strains have different evolutionary trajectories.

Natural selection tends to favor the preservation of S substitutions and transitions in nucleotides, whereas NS substitutions and transversions are generally deleterious mutations that are purged during evolution [19]. The N/S ratios of *S* and *Nsp1* genes were significantly higher than those of other genes, indicating that they are still undergoing rapid evolution and have not yet fully adapted to the host. To further understand the mutation patterns of these genes, we analyzed the mutation types in each site and observed that adenine had the highest substitution frequency (52.11%), followed by cytosine (31.13%). Similar to the NS/S ratio, the transition-to-transversion ratios of *S* (0.672) and *Nsp1* (0.806) were lower than the normal value. The high transversion rates of *S* and *Nsp1* genes helped to explain why the NS/S ratios of these genes were higher than those of other ORFs and confirmed that *S* and *Nsp1* had rapidly mutated.

## 4. Discussion

At present, most Asian countries, including China, have entered a new stage of epidemic prevention and control, with herd immunity forming and people’s lives gradually returning to normal. However, the impact of SARS-CoV-2 cannot be underestimated. In this study, we obtained 92 SARS-CoV-2 genome sequences among 241 cases collected from three countries in South and Southeast Asia from late 2021 to early 2022. We have reported cases with clear epidemic characteristics over time and provided fundamental data for in-depth analysis of the molecular epidemiology and transmission dynamics of SARS-CoV-2 in these regions.

Our results showed that the detection rate of Omicron in positive patients in the Philippines, Pakistan, and Malaysia was not high in November–December 2021. However, within just one month, by January 2022, Omicron emerged as the dominant strain, with a significantly higher detection rate, and subtype BA.2 was more prevalent than subtype BA.1 was. Recent studies have estimated the transmissibility of SARS-CoV-2 strains and found that the early original strain had an R0 of 2–3, whereas the Delta strain has an R0 of 6–7, the Omicron BA.1 variant has an R0 of 7–8, and the BA.2 variant has an R0 as high as 9.1 [5,20]. In this context, long-term epidemiological monitoring is crucial for early detection, decision making, and the response to dangerous SARS-CoV-2 variants.

Despite the complex and challenging nature of tracking the geographical spread of viruses, our study revealed some patterns in the molecular epidemiology and transmission dynamics of SARS-CoV-2 in South and Southeast Asia. By searching for the nearest neighbors of isolated strains, we found that the study isolates were closely related to isolates from England and USA. This relationship may be associated with the relatively early lifting of travel restrictions in these countries during the COVID-19 pandemic. Notably, the BA.2 variant isolated in the Philippines clustered with strains from multiple regions, suggesting the possibility of multiple introductions. South and Southeast Asia have large populations, with some countries such as Singapore and Malaysia having relatively well-developed healthcare systems, whereas others, such as India, Pakistan, and the Philippines, have much less-developed healthcare systems. Therefore, in addition to the common threats of the variants faced by other countries, resource shortages and low vaccination rates have exacerbated the challenges posed by the COVID-19 pandemic in these regions. Therefore, in the event of future pandemics, appropriate travel restrictions may still be necessary in South Asia and Southeast Asia to restrain the spread of the epidemic in its early stages.

It is worth noting that compared to the Delta strain, the Omicron strain has distinct mutation hotspots, with most SNPs being concentrated in the *S*, *Nsp1*, and *Nsp6* genes, and the NS/S ratios of these three genes are significantly higher than those of other ORFs, indicating a prominent contribution to the altered infection characteristics of Omicron. The mutations in the *S* gene are mainly located in the RBD domain, which may directly affect the binding of Omicron to host receptors and the action of existing neutralizing antibodies [4]. Nsp1 is the first protein to be expressed by SARS-CoV-2 after infection and has been found to broadly alter cellular gene expression as a protein translation inhibitor [21,22]. Nsp6 is a non-structural protein that promotes the formation of viral factories. A recent study has demonstrated that mutations in *Nsp6* underlie the reduced pathogenicity of Omicron [23]. Based on the existing evidence, we speculate that the strong infectivity and weak pathogenicity of the Omicron variants currently circulating in South and Southeast Asia are mainly due to functional mutations in the *S*, *Nsp1*, and *Nsp6* genes. This study had some limitations, including a limited sample size, which may have led to a bias in the epidemiological characteristics in a few countries and, therefore, cannot accurately represent the entirety of South and Southeast Asia. Nevertheless, the study still revealed the spread of the new variant in these regions, providing a reference for addressing the ongoing SARS-CoV-2 evolution in this area.

## 5. Conclusions

Overall, the emergence of new SARS-CoV-2 variants and waves of epidemics has brought severe challenges to the world. We reported the variant transitions in Pakistan, the Philippines, and Malaysia from late 2021 to early 2022 and the distinctive mutation characteristics of Omicron variants. The study results can help predict the evolutionary direction of new variants and determine whether current surveillance strategies and responses are appropriate. Because of the high population density and limited healthcare resources, the governments in South and Southeast Asian countries need to strengthen their public health systems, improve their healthcare levels and awareness, and enhance international cooperation to better cope with future pandemic challenges.

## Figures and Tables

**Table 1:** High-frequency substitution sites in Omicron and Delta.

Strain	ORF	Frequency of SNPs
Omicron	*ORF1ab*	G670T (51), C9866T (49), C10029T (63), A10449C (62), C17410T (51), A18163G (56)
	*S*	A22578G (55), G22882T (57), A22992G (57), A23013C (57), A23040G (57), A23055G (57), A23063T (53), C23075T (53), C23525T (48), A23854C (61), G23948T (61), A24424T (57), A24469T (54)
	*ORF3a*	C25810T (49), C26060T (51)
	*E*	C26270T (56)
	*M*	A26709G (57)
	*ORF6*	A27383T (48), C27384T (48)
	*N*	C28311T (60), A29510C (51)
Delta	*ORF1ab*	C14408T (9), A15451G (10), C16466T (10)
	*S*	G22917T (10), A23403G (9), A24410G (10)
	*ORF3a*	C25469T (10)
	*M*	C26767T (9)
	*ORF7a*	C27638T (9), C27752T (10)
	*N*	A28461G (9), A28881C (8), G29402T (10)
All	*S*	A22995C (58 (Omicron), 10 (Delta))

## Data Availability

The raw sequence data reported in this paper were deposited in the Genome Sequence Archive in National Genomics Data Center, China National Center for Bioinformation/Beijing Institute of Genomics, Chinese Academy of Sciences (BioProject accession number: PRJCA016695), which are publicly accessible via https://ngdc.cncb.ac.cn/gsa.

## Supplementary Materials

The following supporting information can be downloaded at: https://www.mdpi.com/article/10.3390/v15061355/s1. Figure S1: The distribution of Ct values of positive swabs from COVID-19 samples; Table S1: Sequencing information of COVID-19 samples; Table S2: Mutation types at each site in all samples.
