# Supplementary material for: Genomic Surveillance of SARS-CoV-2 Variants That Emerged in South and Southeast Asia during Early 2022
Source: Viruses. 2023 Jun 12;15(6):1355. doi: 10.3390/v15061355 (PMC10301783; doi:10.3390/v15061355)
Supplement: Supplementary file 1 [file viruses-15-01355-s001.zip › Figure S1.pdf]

Supplementary Material

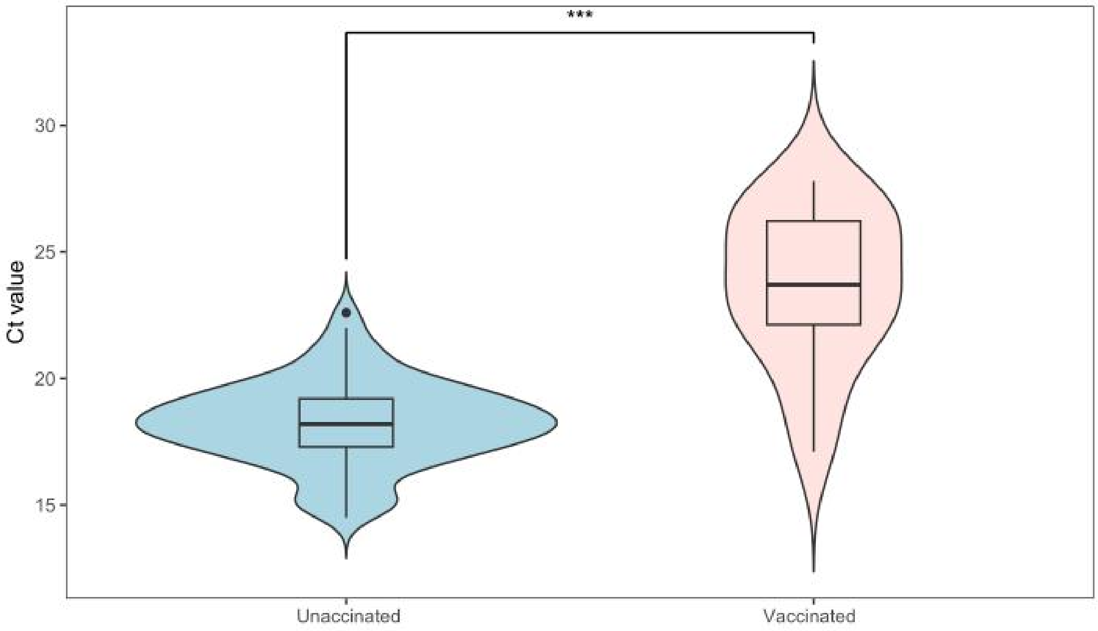

Figure S1. Distribution of Ct values of positive swabs from unaccinated and vaccinated COVID-19 samples. The graph shows the distribution of Ct values, with the boxplot displaying the median, upper and lower quartiles, and whiskers extending to the maximum and minimum values. The violin plot displays the distribution of specific Ct values. Ct values between the unvaccinated and vaccinated groups are statistically significant(t-test, *** $p < 0.001$).
